# Satiating Effect of a Ketogenic Diet and Its Impact on Muscle Improvement and Oxidation State in Multiple Sclerosis Patients

**DOI:** 10.3390/nu11051156

**Published:** 2019-05-23

**Authors:** María Benlloch, María Mar López-Rodríguez, María Cuerda-Ballester, Eraci Drehmer, Sandra Carrera, Jose Joaquin Ceron, Asta Tvarijonaviciute, Javier Chirivella, David Fernández-García, Jose Enrique de la Rubia Ortí

**Affiliations:** 1 Department of Nursing, Catholic University San Vicente Mártir, 46001 Valencia, Spain; maria.benlloch@ucv.es (M.B.); m.cuerda@hotmail.com (M.C.-B.); david.fernandez@ucv.es (D.F.-G.); joseenrique.delarubi@ucv.es (J.E.d.l.R.O.); 2 Department of Nursing, University of Almería, 04120 Almería, Spain; 3 Department of Physical Activity and Sports Sciences, Catholic University San Vicente Martir, 46001 Valencia, Spain; eraci.drehmer@ucv.es; 4 Department of Health Sciences, Catholic University San Vicente Martir, 46001 Valencia, Spain; sandracarrerajulia@gmail.com; 5 Interdisciplinary Laboratory of Clinical Analysis, Campus of Excellence Mare Nostrum, University of Murcia, 30100 Murcia, Spain; jjceron@um.es (J.J.C.); asta@um.es (A.T.); 6 Fundación FIVAN, 46005 Valencia, Spain; xiri@mac.com

**Keywords:** satiety, ketogenic diet, multiple sclerosis, β-hydroxybutyrate, ghrelin, paraoxonase 1

## Abstract

Background: It was previously established that Multiple sclerosis (MS) generates energy alterations at the mitochondrial level related to the loss of muscle mass. Ketone bodies, mainly beta-hydroxybutyrate (BHB), re-establish this energy alteration causing satiety, changes in body composition and a decrease in hormone-dependant hunger, such as ghrelin. The aim of this study was to establish possible improvements in body composition and the level of oxidation in patients with MS, by means of the satiating effect of a ketogenic diet. Methods: A pilot study was carried out with 27 MS patients who were given a Mediterranean isocaloric and ketogenic diet for 4 months. Anthropometric measurements, as well as satiety and hunger perception (VAS scale), were taken. In addition, BHB and paraoxonase 1 (PON1), as an oxidation marker, were measured by spectrophotometric automated assays, and ghrelin was determined by an enzyme immunoassay in the serum. All measurements were taken before and after the intervention. Results: A significant increase in satiety perception at lunch and dinner and of BHB in the blood was obtained. Hunger perception decreased significantly at lunch and dinner with similar levels of ghrelin. In addition, an important increase in lean mass and PON1 was observed. To our knowledge, this is the first study addressing improvements in body composition, oxidation state and metabolism in MS patients, based on the satiating effect of a Mediterranean isocaloric diet. Conclusion: A ketogenic diet increases lean mass and decreases inflammation and oxidation possibly as a consequence of an increase in satiety and decrease in hunger in MS patients.

## 1. Introduction

Multiple sclerosis (MS) is a neurodegenerative disease of an autoimmune nature that produces inflammation and demyelination on the sheath of neurons, therefore altering energy activity of axons on a mitochondrial level [1,2,3]. This leads to a degenerative process due to the lack of trophic support provided by myelin. Some of the physical consequences include muscle loss or changes [4,5], directly associated with a deterioration in the complex I enzyme of the mitochondrial respiratory chain [4]. This deterioration involves lower muscle mitochondrial capacities, 40% less than a healthy person.

Ketone bodies have neuroprotective effects in energy alterations at a mitochondrial level which have been demonstrated in several neurological disorders, such as epilepsy [6,7], Parkinson’s disease [8] or Alzheimer’s disease [9]. This neuroprotection is based on different mechanisms. On the one hand, the beta-hydroxybutyrate (BHB) ketone body is part of a group of hydroxycarboxylic acids able to activate certain hydroxy-carboxylic acid (HCA) receptors that help to maintain homeostasis by changing metabolic and dietary conditions and being able to be used in metabolic and inflammatory disorders. On the other hand, ketogenic diets could cause changes in the way the brain’s energy levels are used, increasing the medium-chain triglycerides (MCT)1 and MCT4 transporters and decreasing the glucose transporter 1 (GLUT1) transporter, which are responsible for transporting ketone bodies and glucose, respectively, through the blood-brain barrier (BBB), especially in the hippocampus and the prefrontal cortex [10], regions which are deteriorated in MS patients [11,12]. Furthermore, ketone bodies are involved in other mechanisms related to muscle function. Thus, BHB has pleiotropic effects that convert it into a metabolite that regulates oxidation stress and inflammation [13], being a prime and/or characteristic factor of conditions related to muscle atrophy. Therefore, ketone bodies show anabolic and anti-catabolic activity in skeletal muscle [14,15,16], especially in BHB activity [17]. Finally, ketone bodies restore the activity of complex 1 of the electron transport chain [18] that is deteriorated in MS.

Previous studies relate ketone bodies to an increase in satiety [19,20]. The mechanism on which this satiety effect is based is complex and depends on the relation that is established with several hormones and metabolites, mainly on a peripheral level. Nonetheless, the effect of fat oxidation by astrocytes at the brain level seems particularly relevant. This oxidation produces ketone bodies that activate the ventromedial nucleus of the hypothalamus, which is directly related to satiety, and which varies throughout the day according to the intake of these fats [21]. As a consequence of this satiating effect, changes in body composition characterised by weight loss are produced [22,23], related to lower resistance to insulin and a low atherogenic lipid panel [24]. Meanwhile, an increase in lean mass is shown; therefore, weight loss would be mainly based on a lower amount of body fat [25,26].

Medium-chain triglycerides (MCTs) made up of medium-chain fatty acids (MCFAs) are the most important source for ketone bodies. Amongst the different food containing MCTs, coconut oil is most likely the nutrient with the most MCTs in its composition, as its percentage of MCFAs exceeds 50% (49% lauric acid, 8% caprylic acid, 7% decanoic acid and a small percentage of hexanoic acid) [27]. MCFAs have a high oxidation rate to obtain energy, avoiding storing it in fatty tissue, and enhancing further energy use [28] and weight loss without long-term recovery. This effect would be even more effective and beneficial if ketogenesis was based on a Mediterranean diet [29], which can also contribute to the satiating effect, if it contained nutrients rich in fibre [30], proteins [31] and complex sugars, due to its ability to control the glycaemic index [32,33]. Finally, the number of meals could be important, as a diet distributed over five meals a day has shown to be more satiating [34,35].

In the same sense as the satiating effect, the feeling of hunger also contributes to a correct energy balance. This feeling depends on several metabolites and hormones, amongst which is ghrelin. This hormone is mainly produced in the stomach [36] and can cross the BBB [37], joining its specific receptor (GHS1a) and finally activating the Y neuropeptide, thus being a powerful appetite stimulator [38]. Its secretion rhythm is circadian, which increases with fasting and the temporary time before meals. Regarding its relationship with ketone bodies, a rapid increase in them causes an immediate decrease in the secretion of ghrelin, as well as appetite and the desire to eat [39]. However, a change in hormone secretion behaviour has been observed in the long term, both for fasting and postprandial, maintaining similar levels despite a decrease in weight and increase in BHB.

Weight loss diets can be related to dietary imbalances that can cause reactive oxygen species (ROS) [40] related to cell damage, accelerated ageing and neurodegenerative diseases, such as Alzheimer’s or Parkinson’s disease [41]. Nonetheless, ketogenic diets have shown improvements in metabolic and inflammatory markers, including lipid markers, glycated haemoglobin (HbA1c) or high-sensitive PCR [42], related to an increase in the total antioxidant status in the blood. Paraoxonases (PONs) are a family of enzymes consisting of PON1, PON2 and PON3. PON1 inhibits low-density lipoprotein (LDL) oxidation, thus preventing the production of cytokines, inflammatory mediators and cell adhesion molecules, thereby decreasing the inflammatory response and LDL deposition in blood vessels [43]. Previous studies have highlighted a correlation between the decrease in PON1 and the progress of some diseases with high oxidative stress and inflammation. All these indications make it an inflammatory marker, being especially efficient to establish metabolic improvements. A decrease in its activity has been observed in patients with MS. Based on the aforementioned, the aim of the study was to determine the possible improvements in body composition, metabolic markers, such as ketone bodies (BHB) and ghrelin, and in antioxidant markers, such as PON1 in MS patients, through the satiating effect of an isocaloric Mediterranean diet rich in fats with a high MCT content.

## 2. Materials and Methods

A prospective, mixed and quasi-experimental pilot study was conducted by means of a clinical trial.

### 2.1. Subjects

In order to obtain the population sample, we contacted the main state-wide MS associations, which informed their members about the nature of the study. The following selection criteria were applied to the 35 people interested in participating in the study: patients over 18 years of age diagnosed with MS at least 6 months ago and treated with glatiramer acetate and interferon beta. Moreover, the exclusion criteria included: pregnant or breastfeeding women, patients with tracheotomy, stoma or with short bowel syndrome, patients with dementia, evidence of alcohol or drug abuse, with myocardial infarction, heart failure, cardiac dysrhythmia, symptoms of angina or other heart conditions, patients with kidney conditions with creatinine levels two times higher than normal markers, patients with elevated liver markers three times higher than normal or with chronic liver disease, patients with hyperthyroidism, patients with acromegaly, patients with polycystic ovary syndrome or MS patients who were included in other researches with experimental drugs or treatment.

### 2.2. Statistical Analysis

Statistical analysis was performed with the SPSS v.23 (IBM Corporation, Armonk, NY, USA) tool. The first step aimed to estimate the distribution of the variables investigated through statistical methods for the assessment of normality, including the Kolmogorov-Smirnov Test. This analysis demonstrated the non-normal distribution of all the scale variables studied. Therefore, the Wilcoxon signed-rank test was used to assess changes before and after the intake of the study diet. Categorical data were analysed with a chi-square test. Finally, a two-tailed Spearman’s test was employed for correlation analysis. A *p*-value below 0.05 was considered significant. Data are presented as mean ± standard deviation or the number of patients and percentage.

### 2.3. Procedure

Once the sample was obtained, the volunteers and their families received detailed information on the study. They were also given instructions to not change their lifestyle habits throughout the 4 months of the duration of the study. They were provided with information on the characteristics of a ketogenic diet and their obligation to follow it over the 4-month period. Once these requirements were accepted, the participants signed an informed consent form. In order to verify that the patients complied with the treatment, weekly telephone calls were made in which they were asked about any doubts and problems in following the diet.

### 2.4. Intervention

Once each participant had been assessed, a ketogenic diet was designed by using the nutritional-dietary software “Nutrition and Health” version 2.0^®^ (University of Granada, Granada, Spain). This diet was given for a 4-month period and was adapted to the individual characteristics of each participant. It consisted of an isocaloric diet with 5 meals a day: breakfast, mid-morning snack, lunch, afternoon snack and dinner. The percentage distribution of the 3 main macronutrients with respect to the total caloric value of the diet was as follows: 20% proteins, 40% carbohydrates and 40% lipids. The diet was based on promoting healthy food and avoiding ultra-processed products. Thus, foods with a high content of rapidly-digested simple sugars (dairy desserts, juices, chocolate bars and soft drinks), unhealthy fats (cold meats and pastries) and with a high level of salt and additives (snacks) were avoided. In addition, the consumption of alcohol, both fermented and distilled, was not considered. Foods rich in proteins of a high biological value of animal origin were included, such as lean meat, white and oily fish, eggs, seafood and dairy and healthy derivatives (yoghurt and cheese). Proteins of vegetable origin cooked in a healthy way and roasted or raw nuts without salt were also included. In order to guarantee sufficient vitamins, minerals and fibre, the consumption of low glycaemic vegetables and fruits and slowly-digested complex carbohydrates were included, preferably a wholemeal whenever possible (rice, pasta, legumes, tubers) and cooked in a healthy way.

Regarding the diet’s lipid panel, which corresponds to 20% of the total caloric value, it was distributed as follows: 65.69% saturated fatty acids, 19.64% monounsaturated fatty acids and 14.77% polyunsaturated fatty acids. In addition, the diet provided a total daily amount of 232.41 mg of cholesterol and 38.74 g of fibre. Finally, with the aim of achieving a ketogenic state, a total of 60 mL of coconut oil was provided on a daily basis (30 mL of coconut oil for breakfast and 30 mL for lunch). The lipid panel of the 60 mL of coconut oil was as follows: 91.84% saturated fatty acids, 6.23% monounsaturated fatty acids and 1.93% de polyunsaturated fatty acids. This fatty acid intake was included in the isocaloric diet and was adapted to an adult’s nutritional requirements.

### 2.5. Measurements

The following measurements were taken before and after the 4-month intervention, in the same conditions and by the same researcher assigned to the study.

- Nutritional and dietary anamnesis: Just before starting the study, a nutritional and dietary anamnesis was carried out for each patient with the Food Frequency Questionnaire (FFQ) [44]. This tool made it possible to determine how often foods belonging to different groups were consumed: dairy products, vegetables, fruits, juices, nuts, meat, fish, seafood, eggs, tubers, rice, legumes, pasta, cold meats and sausages, snacks, pastries and biscuits, chocolate bars, soft drinks, fermented alcohol and distilled alcohol.
- Body composition: Measurements related to weight, height, skin folds and body perimeters and diameters were taken using the Faulkner method, taking into account the protocol currently established by The International Society for the Advancement of Kinanthropometry (ISAK). Furthermore, we collaborated with an ISAK level 2 certified anthropometrist [45]. A portable clinical scale, SECA model, with a 150–200 kg capacity and 100 g precision was used, a stadiometer, model SECA 220 Hamburg, Germany with 0.1 cm precision, a mechanical skinfold caliper, model Holtain LTD Crymych UK with a 0.2 mm precision and measurement range from 0 to 48 mm, a dermographic pencil, a metal, inextensible and narrow anthropometric tape, model Lufkin W606PM with 0.2 mm precision and a bicondylar pachymeter to measure the diameter of small bones, model Holtain, with 1 mm precision and measuring range from 0 to 48 mm.
- Blood test and marker analysis: A blood test was carried out at 9 a.m. on an empty stomach, then the serum was separated from the plasma after centrifuging the samples. The BHB levels were measured with a commercial kit (Randox Laboratories, Crumlin, UK) and PON1 activity by using 4-Nitrophenyl acetate [46]. In both cases, an automated clinical biochemistry analyser (Olympus A 400, Tokyo, Japan) was used. Ghrelin was quantified by means of a commercial method, ELISA kit (BioVendor, Asheville, NC, USA).
- Appetite Assessment: Participants completed an appetite questionnaire for one whole day, immediately before eating a meal and again after 2 h (breakfast, lunch and dinner). The appetite questionnaire used a visual analogue scale (VAS), which has been found to be a reliable and valid tool for appetite assessment [47]. This questionnaire included eight questions followed by 10 cm horizontal lines, where 0 represented “sensation not felt at all” and 10 represented “sensation strongly felt”. Subjects were asked to mark the line at the point which corresponded to how they were feeling at that particular time. The questionnaire was developed by Parker et al. [48] to assess human appetite. The distance from the beginning of the line to the participants’ mark was measured from the left-hand side.

### 2.6. Ethical Concerns

The study was developed in accordance with the Declaration of Helsinki [49] with the prior approval of the protocol by the University of Valencia Human Research Committee of the Experimental Research Ethics Committee (procedure number H1512345043343). Participants were provided with written informed consent, after being informed of the nature of the study, as well as all the procedures to which they would be subjected in it.

## 3. Results

This study analysed a sample of 27 MS patients with a comparable proportion of patients of the different types of MS accepted at present and a mean age of 44.56 years (Table 1).

### 3.1. Dietary Habits of the Study Population

According to this study’s data, the study population’s dietary habits before the intervention were based on a high intake of simple sugars (dairy desserts, juices, pastries, chocolate bars and soft drinks) and a low intake of refined complex carbohydrates (rice and pasta). In addition, fruit and vegetable intake was low, therefore providing little fibre to their diet. Regarding fats, they mainly originated from cold meats, pastries, chocolate bars and snacks. Their diets were not rich in proteins (Table 2).

### 3.2. Change in Satiety Perception and BHB Production

Once the intervention had finished, a significant change in the increase in satiety perception before and after lunch and dinner was observed. However, this variation was not observed at breakfast. BHB levels in the blood significantly increased (Table 3).

### 3.3. Percentage Changes in Fat and Muscle and PON1 Levels

**Figure 1:**
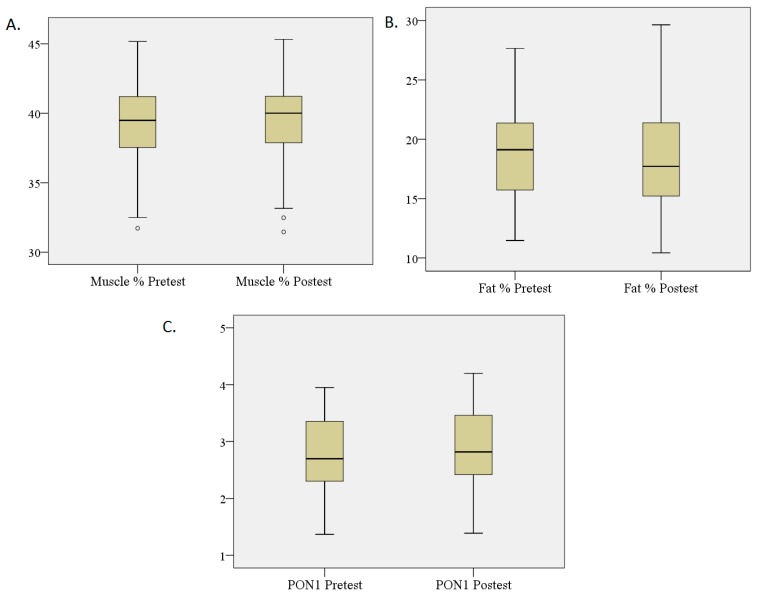
Changes in muscle and fat percentage and PON1 (paraoxonase 1) levels in serum. (**A**) *N* = 27; Body composition measurements were taken using the Faulkner method; Wilcoxon signed-rank test showed a significant increase in muscle mass (*p* = 0.003). (**B**) *N* = 27; Body composition measurements were taken using the Faulkner method; Wilcoxon signed-rank test showed a significant decrease in fat mass (*p* = 0.000). (**C**) *N* = 27; The PON1 activity was measured by using 4-Nitrophenyl acetate; Wilcoxon signed-rank test showed a significant increase in PON1 (*p* = 0.000).

Regarding body composition, a change characterised by a significant increase in lean mass, as well as a significant decrease in fat mass, was observed. Likewise, a significant increase of PON1 in the blood was found (Table 3, Figure 1).

### 3.4. Changes in Hunger Perception and Ghrelin Secretion

After the intervention, hunger perception showed lower scores before and after lunch and dinner. Nonetheless, this change was not observed at breakfast time, where perception was similar to that of before the study. Regarding hunger perception assessed through the blood values of ghrelin, no changes were observed after treatment (Table 3).

## 4. Discussion

Due to pathogenesis based mainly on energy alteration at a mitochondrial level, patients with MS express a worsening of physical function [4,50], decreasing their muscle mass [51]. An alternative treatment is to restore this mitochondrial activity. For this reason, our study prescribed a ketogenic diet for 4 months. According to literature, this is the first study to address the possible improvements in body composition, oxidation state and metabolism in MS patients, based on the satiating effect of a Mediterranean isocaloric diet rich in fats with a high MCT content.

In general, our results coincide with those of other studies, which indicate that ketone bodies can be a good alternative to improve motor function, not only as a result of their neuroprotective properties analysed by other authors [9,10,11] but also due to the metabolic impact based mainly on the satiating effect related to weight loss with an increase in lean mass [25,26]. In particular, there was a significant increase in BHB in serum after our intervention, alongside an increase in lean mass and a decrease in fat mass, which in both cases were significant. In addition, possibly as a result of an anthropometric profile change, there was a significant increase in PON1 levels, a marker associated with low levels of oxidative stress and inflammation. This could be due to an improvement in the total antioxidant status in the blood, already evidenced especially after intake of certain nutrients, such as fruit and vegetables, present in large quantities in the Mediterranean diet, and precisely by promoting higher production of PON1 in the liver [52]. In this sense, the ketogenesis induced in our study has a Mediterranean diet base. This fact could increase the antioxidant activity, which has already been observed after only a three-week ketogenic diet [53,54]. The changes obtained in our study after a four-month intervention imply a variation in the organism’s preference for ketones as a fuel (especially BHB). This was already observed a few weeks after prescribing a ketogenic diet [55,56].

As previously mentioned, the satiating effect of ketone bodies [19,20] could have a relevant role in improving the aforementioned anthropometric improvements. In this sense, and as indicated in previous research [19,20], this study showed a significant increase in satiety. In addition, this effect also saw a lower hunger perception. Nonetheless, ghrelin, the hormone directly related to hunger [57], maintained levels similar to those that patients had before starting the intervention. This discrepancy could be due to the relationship between BHB secretion and the hormone. In accordance with our results, another study [58] showed how ghrelin levels do not vary in the blood if ketone body administration is in the long term, specifically 8 weeks, despite a decrease in weight and increase of BHB. Something similar could be observed when a low carbohydrate ketogenic diet was prescribed for 12 weeks, as there were no variations in ghrelin when fasting, although there was weight loss [59].

After our intervention, changes in satiety and hunger were before and after lunch and dinner. This was not the case with breakfast. The explanation of these results could be based on the hypothesis that the production of both molecules is related in the short term, and during the day, depends on meals. Recently, a significant decrease in ghrelin in the blood has been shown between 2 and 4 h after timely drinking a ketone ester drink, with a peak increase of BHB in the blood after 60 min. This was also associated with an immediate decrease in appetite and the desire to eat after this period of time [39]. In our study, although the levels of ketone bodies and ghrelin were not measured in the different meals to avoid adding stress to the individuals involved in the study, we could say that, based on the aforementioned, due to the use of coconut oil at breakfast and lunch, the highest levels of ketone bodies would be given at lunch and dinner. The same applied to the lowest levels of ghrelin. Therefore, this would explain the feeling of satiety and lack of hunger before and after both meals. In this sense, by not using coconut oil for dinner and after not eating for 8 h, the highest levels of ghrelin in the blood would most likely be produced just before breakfast on an empty stomach. Regarding BHB and its relation to the lack of satiety before and after breakfast, despite an increase in BHB after treatment, it is possibly due to the organism adapting itself to use that source of energy [55,56] that the impact on satiety does not occur before and after breakfast. The peaks after coconut oil intake at breakfast and lunch caused satiety at lunch and dinner. Another aspect that could have an influence on the differences in satiety and hunger observed, among the three main meals, are the changes in dietary habits and nutrients. Regarding dietary habits, patients mainly had four meals a day before the study. However, patients had five meals a day during the intervention. This could have had an influence on satiety and hunger, as smaller and more regular meals (five per day) are related to less sudden changes in blood sugar and lipemia, as well as an improvement in regulating appetite and satiety sensation [34,35].

**Figure 2:**
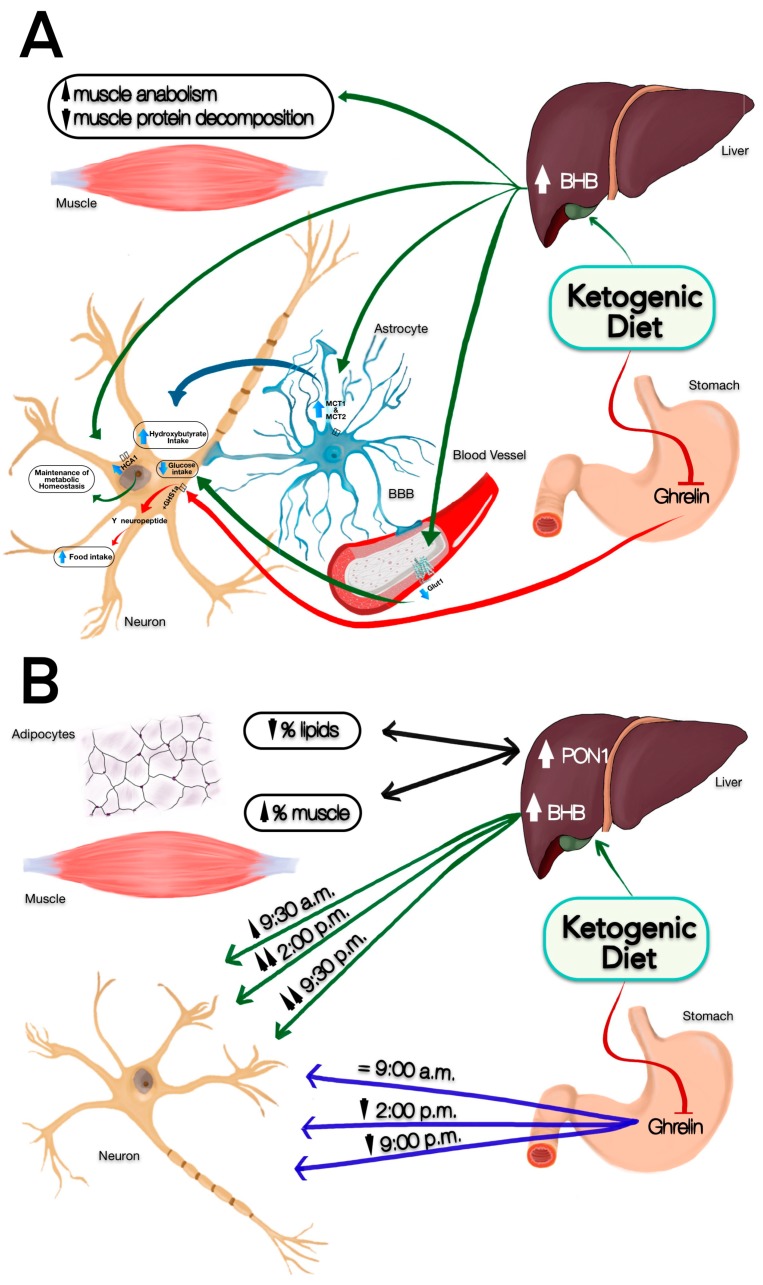
The effects of the ketogenic diet on different organs. (**A**) The beta-hydroxybutyrate (BHB) ketone body changes the way energy is used in the brain, increasing medium-chain triglycerides (MCT)1 and MCT4 in the astrocytes, which are part of the blood-brain barrier (BBB) and decreasing glucose transporter 1 (GLUT1) of the blood vessels of the BBB, being responsible for transporting ketone bodies and glucose, respectively, to the Central Nervous System (CNS). Ketone bodies have anabolic and anti-catabolic activity in the skeletal muscle. Finally, ghrelin joins its specific receptor (GHS1a) within the CNS, activating the Y neuropeptide. (**B**) Possible effects of the ketogenic diet on different organs y molecules (PON1). After the intervention, an increase in BHB production was observed. In addition, the increased perception of satiety during lunch and dinner raises the possibility that this increase will be greater in these temporal intervals (2:00 p.m and 9.30 p.m) depending on the time of administration of coconut oil. Regarding ghrelin, fasting levels are maintained as before the start of the intervention. However, depending on the interaction with the production of ketonic bodies already described, the production could decrease coinciding with the increase of BHB (2:00 p.m and 9.30 p.m). These two associated processes may explain the increase in muscle and the decrease in fat. Both aspects would be related to a better metabolic profile, evidenced by the higher production of PON1 in the liver as an anti-inflammatory marker.

In relation to food consumption, alterations in the glycaemic metabolism have been observed in MS patients, related to damage at the mitochondrial level [60] and excessive production of ROS and inflammation, helping to slow down the progression of the disease [61,62,63]. In addition, fast-digesting simple sugars in the short- and long-term favour appetite and increase intake [32,33], as a rise in insulin leads to an increase in hunger and a decrease in satiety [64] related to a rise in the glycaemic index [65,66]. As a result, this study performed a nutritional change, going from consuming a high intake of carbohydrates, mainly simple sugars, to consuming complex carbohydrates and healthy fats. This would regulate the glycaemic index and, as a result, decrease hunger and increase satiety throughout the day [67,68]. Furthermore, a larger amount of fibre in the diet, in relation to the diet before the study, would favour the regulation of glucose levels and the satiating effect [30], thus explaining the obtained results. Finally, our intervention also included a higher amount of protein than previously consumed and balanced with respect to the other nutrients, which could also contribute to decreasing hunger and increasing satiety [69,70]. In short, comparing the effects of the ketogenic diet on the different organs described in the introduction (Figure 2A), our results after the discussion suggest the contributions that can be seen in Figure 2B.

Despite the fact that our results show evidence of improvement related to the disease and the production of markers related to the perception of satiety and hunger after prescribing a ketogenic diet, the study has a series of limitations. Among such limitations, in addition to a small sample leading to relatively high standard deviations, BHB and ghrelin measurements were not taken during the day. Therefore, future research should include a larger sample. Furthermore, BHB and ghrelin should be measured during the day, in order to better understand the interaction between the variation levels with the perception of satiety and hunger. Finally, it is necessary to study further the behaviour and role of other molecules and parameters directly related to hunger and satiety, highlighting the glycaemic index.

## 5. Conclusions

After the intervention with MS patients had taken place, an increase in lean mass was observed, alongside fat mass loss. These changes could be related to patients’ metabolic profile improvements, evidenced by an increase in PON levels associated with less oxidation and inflammation. Furthermore, a satiating effect was found, alongside a lower feeling of hunger, at lunch and dinner, which could be associated with an increase in BHB levels and changes in dietary habits. These results indicate that obtaining ketogenesis by consuming a Mediterranean diet rich in MCT fats produces a satiating effect, possibly related to metabolic and anthropometric changes that have a positive impact on the clinical evolution of MS. Consequently, this type of diet may represent a therapeutic alternative by supplementing the pharmacological treatment. However, new studies would be required to confirm the conclusions drawn and the mechanisms proposed.

## Figures and Tables

**Table 1:** Sociodemographic characteristics of the study population.

Measure		Frequency	%
MS Type	Primary progressive MS	1	3.7%
	Relapsing-remitting MS	20	74.1%
	Secondary progressive MS	6	22.2%
Gender	Men	5	18.5%
	Women	22	81.5%
		**Mean**	**SD**
Age (years)		44.56	11.27
Time from MS Diagnosis (years)		12	10

MS: Multiple sclerosis. SD: Standard Deviation.

**Table 2:** Dietary habits of the study population prior to the intervention.

Measure	Mean	SD
No. of meals a day	4.00	0.83
No. of monthly intakes of main nutrients		
Dairy	16.81	12.89
Cheeses	11.37	9.86
Dairy desserts	0.78	2.49
Vegetables	19.26	8.81
Fruit	22.67	8.52
Juice	9.93	12.02
Nuts	14.89	10.23
Meat	12.37	6.97
Fish	8.07	4.59
Seafood	3.37	3.25
Eggs	10.22	5.24
Tubers	10.22	5.56
Rice	7.56	3.39
Legumes	6.22	4.59
Pasta	6.52	4.17
Cold meats	14.74	10.66
Snacks	2.89	3.89
Pastries	12.41	11.19
Chocolate bars	10.19	10.50
Soft drinks	5.81	8.87
Fermented alcohol	6.15	7.39
Distilled alcohol	0.15	0.46

SD: Standard deviation.

**Table 3:** Changes in satiety and hunger, muscle and fat, and BHB (beta-hydroxybutyrate), PON1 (paraoxonase 1) and ghrelin levels in serum.

Measure	Pre-Test		Post-Test		Z	*p*
	Mean	SD	Mean	SD		
Before breakfast satiety	4.44	3.06	5.15	2.63	–1.143	0.253
After breakfast satiety	5.38	2.76	6.64	2.76	–1.480	0.139
Before lunch satiety	3.26	3.16	6.22	2.58	–3.387	0.001 *
After lunch satiety	4.56	2.75	8.07	1.72	–3.802	0.000 *
Before dinner satiety	3.90	2.92	5.80	2.71	–2.800	0.005 *
After dinner satiety	5.46	2.13	7.89	2.15	–3.876	0.000 *
BHB (Mmol/L)	0.06	0.04	0.10	0.10	–2.005	0.045 *
Fat %	19.53	3.78	17.74	3.32	–4.421	0.000 *
Muscle %	39.39	2.88	40.22	2.86	–2.955	0.003 *
PON1 (UI/L)	2.67	0.62	2.92	0.68	–3.722	0.000 *
Hunger before breakfast	3.27	2.17	3.14	3.35	–0.622	0.534
Hunger after breakfast	2.88	2.38	2.25	1.93	–1.677	0.094
Hunger before lunch	6.46	2.13	2.15	2.37	–4.306	0.000 *
Hunger after lunch	5.38	2.27	1.02	1.80	–4.346	0.000 *
Hunger before dinner	5.59	2.24	2.54	2.99	–4.077	0.000 *
Hunger after dinner	3.82	2.66	0.91	1.71	–3.744	0.000 *
Ghrelin (pg/mL)	24.04	36.75	24.97	48.94	–0.216	0.829

SD: Standard Deviation; Z: Wilcoxon signed-rank test; BHB: beta-hydroxybutyrate; * *p* < 0.005.
